# Selfing Shapes Fixation of a Mutant Allele Under Flux Equilibrium

**DOI:** 10.1093/gbe/evae261

**Published:** 2024-12-04

**Authors:** Yu Xiao, Yan-Wen Lv, Zi-Yun Wang, Chao Wu, Zi-Han He, Xin-Sheng Hu

**Affiliations:** College of Forestry and Landscape Architecture, South China Agricultural University, Guangzhou 510642, China; Guangdong Key Laboratory for Innovative Development and Utilization of Forest Plant Germplasm, South China Agricultural University, Guangzhou 510642, China; College of Forestry and Landscape Architecture, South China Agricultural University, Guangzhou 510642, China; Guangdong Key Laboratory for Innovative Development and Utilization of Forest Plant Germplasm, South China Agricultural University, Guangzhou 510642, China; College of Forestry and Landscape Architecture, South China Agricultural University, Guangzhou 510642, China; Guangdong Key Laboratory for Innovative Development and Utilization of Forest Plant Germplasm, South China Agricultural University, Guangzhou 510642, China; College of Forestry and Landscape Architecture, South China Agricultural University, Guangzhou 510642, China; Guangdong Key Laboratory for Innovative Development and Utilization of Forest Plant Germplasm, South China Agricultural University, Guangzhou 510642, China; College of Forestry and Landscape Architecture, South China Agricultural University, Guangzhou 510642, China; Guangdong Key Laboratory for Innovative Development and Utilization of Forest Plant Germplasm, South China Agricultural University, Guangzhou 510642, China; College of Forestry and Landscape Architecture, South China Agricultural University, Guangzhou 510642, China; Guangdong Key Laboratory for Innovative Development and Utilization of Forest Plant Germplasm, South China Agricultural University, Guangzhou 510642, China

**Keywords:** mating system, mutation, gametophytic selection, sporophytic selection, genetic load

## Abstract

Sexual reproduction with alternative generations in a life cycle is an important feature in eukaryotic evolution. Partial selfing can regulate the efficacy of purging deleterious alleles in the gametophyte phase and the masking effect in heterozygotes in the sporophyte phase. Here, we develop a new theory to analyze how selfing shapes fixation of a mutant allele that is expressed in the gametophyte or the sporophyte phase only or in two phases. In an infinitely large population, we analyze a critical selfing rate beyond which the mutant allele tends to be fixed under equilibrium between irreversible mutation and selection effects. The critical selfing rate varies with genes expressed in alternative phases. In a finite population with partial self-fertilization, we apply Wright's method to calculate the fixation probability of the mutant allele under flux equilibrium among irreversible mutation, selection, and drift effects and compare it with the fixation probability derived from diffusion model under equilibrium between selection and drift effects. Selfing facilitates fixation of the deleterious allele expressed in the gametophyte phase only but impedes fixation of the deleterious allele expressed in the sporophyte phase only. Selfing facilitates or impedes fixation of the deleterious allele expressed in two phases, depending upon how phase variation in selection occurs in a life cycle. The overall results help to understand the adaptive strategy that sexual reproductive plant species evolve through the joint effects of partial selfing and alternative generations in a life cycle.

**Significance**

This study develops a new theory to show how the joint effects of selfing and alternative generations in plant life cycle shape fixation of a mutant allele. The theory helps to interpret a potentially adaptive strategy that flowering plant species could evolve through partial selfing to regulate fixation of mutations in the gametophyte phase versus the sporophyte phase.

## Introduction

Alternative generations in a plant life cycle provide a biological basis for genes expressed in the gametophyte phase or the sporophyte phase only or in both phases. Ample evidence indicates that many genes are highly expressed in male gametophyte (Honys and Twell 2003, 2004; Borg et al. 2009; Deveshwar et al. 2011) or in female gametophyte (Yu et al. 2005; Ma et al. 2018; Singh et al. 2023). These genes are involved in a wide range of biological functions, such as pollen-related (da Costa-Nunes and Grossniklaus 2003; Wei et al. 2010; Warman et al. 2020; Lobaton et al. 2021) or ovule-related development (Wu et al. 2015; Pathak et al. 2016; Wang et al. 2016; Hou et al. 2021) or both gametophytic and sporophytic development (Arunkumar et al. 2013; Sigel et al. 2018; Somers and Nelms 2023). These expressed genes could be regulated by different ecological and evolutionary mechanisms.

Empirical studies indicate that genes expressed in alternative phases could have unequal evolutionary rates (Arunkumar et al. 2013; Szovenyi et al. 2013, 2014; Beaudry et al. 2020). This potentially arises from natural selection and genetic drift effects (Bedford and Hartl 2009; Price et al. 2022). It is well recognized that gametophytic selection efficiently purges deleterious alleles since all gametes are completely exposed to selection (Delph and Havens 1998; Delph 2019). Sporophytic selection is not as efficient as gametophytic selection because deleterious alleles may be masked from selection in heterozygotes (Charlesworth and Charlesworth 1992). Antagonistic selection between two phases facilitates allelic polymorphisms and hence reduces the fixation probability of a mutant allele. Previous theories have addressed antagonistic selection between haploids and diploids in maintaining polymorphisms (Haldane 1932; Haldane and Jayakar 1963; Damgaard et al. 1994; Immler et al. 2012; Otto et al. 2015). A gene exhibiting differential levels of expression between two phases is potentially under antagonistic selection (Gossmann et al. 2016; Beaudry et al. 2020). To the contrary, synergistic selection between two phases facilitates loss or fixation of a mutant allele and a favorable mutant allele is expected to be rapidly fixed (Charlesworth and Charlesworth 1992; Walsh and Charlesworth 1992). Therefore, given an effective population size (*N_e_*), phase variation in selection could yield unequal fixation rates between genes expressed in alternative generations in a life cycle.

Mating system as a life history trait can regulate gene expression in either the gametophytic phase or the sporophytic phase or in both and hence shapes gene fixation and evolutionary rates (Li et al. 2023a). Although mating system is not considered as one of basic evolutionary forces (selection, mutation, drift, and migration), it interacts with each force and determines the distribution of genotypes within populations (Muyle and Marais 2016; Hu et al. 2021). Thus, the gene fixation process differs in populations with different selfing rates. Here, selfing refers to fertilization of ovules (female gametes) by pollen (male gametes) from the same diploid plant, and the intragametophyte selfing where two gametes from the same haploid gametophyte combine to produce homozygous sporophyte is not considered (Klekowski 1979). Partial selfing can cascade at least two processes to influence gene fixation. One is to reduce effective population size (*N_e_*) and potentially enhance fixation of a mutant allele (Kimura 1962; Caballero and Hill 1992; Glemin 2007). The second process is through the interaction of selfing with selection. Selfing affects the efficacy of gametophytic selection through reducing gametic competitiveness from alien pollen grains (Hu 2015). Selfing also interacts with sporophytic selection through increasing homozygosity. A complete selfing species yields comparable selection efficacies between gametophytic and sporophytic selection since the masking effects on deleterious alleles in heterozygotes are absent (Mazer et al. 2010). These two processes jointly affect fixation of a mutant allele in a partially self-fertilized population (Damgaard 2000). Here, we concentrate on the second process, given an effective population size, and explore how selfing regulates the fixation probability of a mutant allele that is expressed in a single phase or in two phases.

Previous theories in this field go back to the pioneering work by Fisher (1930), Haldane (1927, 1932), Wright (1931, 1937, 1945) and followed by Kimura (1962). Since then, many theories have been developed to extend the assumptions in earlier theories with Galton–Watson branching process for an infinitely large population and the diffusion process for a finite population (Patwa and Wahl 2008). These extensions generally cover two processes: genetic drift and selection. Extensions in the genetic drift process include examination on the effects of fluctuating population sizes (Ewens 1967; Wahl and DeHaan 2004; Parsons and Quince 2007), bottlenecks (Wahl and Gerrish 2001), and population subdivision (Pollak 1966; Barton 1993; Whitlock 2003; Gordo and Campos 2006). Extensions in the selection process include a more complex branching process (Wilke 2003) for advantageous alleles and the linkage effects on deleterious alleles (Barton 1994; Johnson and Barton 2002). Nevertheless, the phase variation in selection in a plant life cycle has not been fully explored in shaping the fixation probability of a mutant allele (Charlesworth 1992; Patwa and Wahl 2008; Waxman 2011; Bessho and Otto 2017; Li et al. 2023b).

We begin by addressing deterministic processes in a large population and analyze how large the selfing rate is required to fix a mutant allele under the equilibrium between irreversible mutation and selection effects. We then apply Wright's method to calculate fixation probability of a mutant allele under phase variation in selection (Wright 1938, 1945, 1969). A stochastic process is considered in a small population, where the steady distribution of the mutant allele frequency is attained under equilibrium among irreversible mutation, selection, and drift effects. The fixation of a mutant allele is derived from the fundamental density distribution of allele frequency and compared with the results derived from the diffusion model under equilibrium between selection and drift effects (Kimura 1962). Monte Carlo (MC) simulations are used to check the theoretical predictions and to illustrate the effects of selfing on fixation of a mutant allele.

## The Theory

### General Assumptions

The model deals with a diploid hermaphrodite plant with alternative generations (gametophyte and sporophyte phases) in a life cycle, which consists of sequential events, including generation of haploid pollen and haploid ovules (or male and female gametes), gametophytic selection, a mixed mating system (selfing and outcrossing), seed formation, mutation, sporophytic selection, and genetic drift. The life cycle is the same as Fig. 1 of Li et al. (2023b) except for including an additional mutation event occurring before sporophyte selection. Assume that a population is completely isolated, so that the effects of gene flow on allele fixation are excluded. Weak selection is considered in both gametophyte and sporophyte phases. For plants with differential fitnesses between males and females in the gametophyte phase (Bull 1978; Immler and Otto 2015), the model allows unequal strengths of selection between pollen (haploid males) and ovules (haploid females). With the mixed mating system, we consider a constant selfing rate *α* and the outcrossing rate 1−*α* in the population. The model does not consider the intragametophyte selfing that occurs in some haploid-dominant ferns (Klekowski 1979). Note that the intergametophytic self-fertilization (e.g. in ferns) refers to the fusion of two gametes from different haploid gametophytes of the same plant, which is also referred to selfing here (Hedrick 1987). The plant species is complete outcrossing when the selfing rate *α* is equal to 0 and completely self-fertilized when the selfing rate *α* is equal to 1. Thus, the model is appropriate for flowering plants (gymnosperms and angiosperms) and is potentially applicable to some pteridophyte species when intragametophyte selfing and asexual reproduction are neglectable (Hedrick 1987). The model is not appropriate for algae, lichens, and bryophytes where asexual reproduction is important and for fungi where the alternation of generations in a life cycle is absent.

**Fig. 1.**
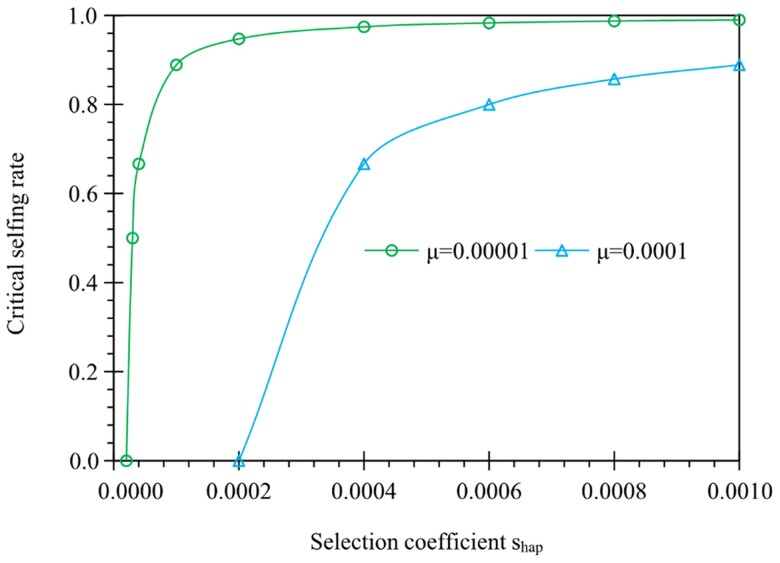
Examples of the critical selfing rate for genes expressed in the gametophyte phase only. Results are derived from equation (2), showing that the critical selfing rate has a negative relationship with the mutation rate ($\mu =10^{-4}$ and 10^−5^) but a positive relationship with the selection coefficient ($s_{\mathrm{hap}}$) increasing from 0.00002 to 0.001 for a deleterious allele.

Consider one nuclear locus with two alleles (*A*, *a*) in a population. Allele *A* is the ancestral type and *a* is the mutant allele. A systematic change in allele frequency is derived according to the sequential events before the occurrence of genetic drift in the life cycle. We follow Wright's idea (1969) to derive genotypic frequencies in a mixed mating system, where the selfing and outcrossing parts are separately calculated and then combined. Two specific scenarios are separately addressed. One is an infinitely large population where the deterministic processes are applied to assess fixation of the mutant allele. The second scenario is a finite population where a random genetic process is applied to analyze fixation of the mutant allele. For genes expressed in both gametophytic and sporophytic phases, we consider two models of phase variation in selection: antagonistic and synergistic selections.

To evaluate the fixation probability of the mutant allele under random genetic drift process, we apply MC simulations to check theoretical predictions. Scripts in C are provided in the Supplementary material online. A subroutine in C from Press et al. (1991) is employed for generating random numbers with uniform distribution *U*[0,1]. For each case, we conduct 10,000 simulation runs with each run reaching the steady-state distribution of allele frequency. The fixation probability of the mutant allele is then calculated. To assess the reliability of the estimates of the fixation probability, we obtain 100 independent datasets for each case, so that mean and standard deviation of the fixation probability are estimated from these replicated datasets.

### Systematic Change in Allele Frequency

Let *p* and *q* (p+q=1) be the frequencies of the ancestral (*A*) and mutant (*a*) alleles, respectively. Let $F_{is}$ be the inbreeding coefficient in the population. The three genotypic frequencies in current adults are expressed as $p_{AA}=p^{2}+pqF_{is}$, $p_{Aa}=2pq(1-F_{is})$, and $p_{aa}=q^{2}+pqF_{is}$. Consider the selfing part, with a probability of *α*. Let the gametic fitness be 1 for allele *A* in both pollen and ovules, and $1-s_{O}$ and $1-s_{P}$ for allele *a* in ovules and pollen, respectively. The mutant allele is deleterious when the selection coefficients are positive ($s_{O}>0$ and $s_{P}>0$) but favorable when negative ($s_{O}<0$ and $s_{P}<0$). The average fitness is 1, $1-\frac{1}{2}s_{O}$, and $1-s_{O}$ in ovules produced by genotypes *AA*, *Aa*, and *aa*, respectively. Similarly, the average fitness is 1, $1-\frac{1}{2}s_{P}$, and $1-s_{P}$ in pollen produced by genotypes *AA*, *Aa*, and *aa*, respectively. After the gametophytic selection and selfing, the genotypic frequencies can be calculated from the genotypic frequencies in the preceding adults.

Consider the outcrossing part, with a probability of 1−α. In the gametophyte phase, the average fitness is $1-s_{O}q$ in ovules and $1-s_{P}q$ in pollen. The genotypic frequencies are calculated after selection in the gametophytic phase and then a random combination between pollen and ovules. Thus, the overall genotypic frequencies are calculated by combining the selfing and outcrossing parts weighted by *α* and 1−α, respectively, in the mixed mating system. For the plant species with sexual divergence in haploid phase, the selection coefficients may be unequal between pollen and ovules ($s_{O}\neq s_{P}$).

Assume that mutation occurs in the sporophyte phase. Note that the systematic change in allele frequency is not altered under weak selection when mutation is assumed to occur in the gametophytic phase. Let *μ* be the mutation rate from allele *A* to *a*, and the reverse mutation is absent (irreversible mutation). The genotypic frequencies are then recalculated. In the sporophyte phase, let the fitness be $W_{AA}=1$ for homozygote *AA*, $W_{Aa}=1+s_{\mathrm{het}}$ for heterozygote *Aa*, and $W_{aa}=1+s_{\mathrm{hom}}$ for homozygote *aa*. The average fitness in the sporophyte phase is calculated by $\bar{W}=W_{AA}p_{AA}^{**}+W_{Aa}p_{Aa}^{**}+W_{aa}p_{aa}^{**}$ where $p_{AA}^{**}$, $p_{Aa}^{**}$ and $p_{aa}^{**}$ are the frequencies of three genotypes after mutation (see Appendix A, Supplementary material online for details). The genotypic frequencies in the next adults are $p_{AA}^{***}=w_{AA}p_{AA}^{**}/\bar{W},\;\;$  $p_{Aa}^{***}=w_{Aa}p_{Aa}^{**}/\bar{W}$, and $p_{aa}^{***}=w_{aa}p_{aa}^{**}/\bar{W}$. After sophisticated algebraic calculations, the systematic change of the mutant allele frequency is derived by $\Delta q=q^{\prime }-q$, where $\;q^{\prime }=p_{aa}^{***}+\frac{1}{2}p_{Aa}^{***}$ is the frequency after sporophytic selection. Previous studies have shown that the population inbreeding coefficient $F_{is}$ is approximated by α/(2−α) under the equilibrium between selfing and outcrossing (Haldane 1924; Wright 1969). Under weak selection, this approximation also holds at equilibrium ($p_{Aa}^{***}=p_{Aa}$) by omitting the effects of selection and mutation from equation (A16) in Appendix A, Supplementary Material online.

From equation (A18) in Appendix A, Supplementary Material online, we obtain:


(1) $$\begin{array}{rl} \Delta q\;= & \;p\mu -\frac{1}{2}pq(1-F_{is})s_{\mathrm{hap}}-pq(q-p)(1-F_{is})s_{\mathrm{het}}\\ & +pq(q+pF_{is})s_{\mathrm{hom}} \end{array}$$

where $s_{\mathrm{hap}}=s_{O}+s_{P}$, a composite selection coefficient from pollen and ovules. Since the coefficients ($s_{O}$ and $s_{P}$) are compounded, the fitness differentiation in pollen (male gametes) and ovules (female gametes) would not affect Δq, given a composite selection coefficient ($s_{\mathrm{hap}}$). The above expression indicates that selfing interacts differently with the gametophytic and sporophytic selection. An influx of mutation facilitates maintenance of the mutant allele.

### Fixation Under Deterministic Processes

In an infinitely large population, we examine the condition of how large a selfing rate is needed to drive the fixation of the mutant allele. Here, we propose a critical selfing rate $\alpha^{*}$ beyond which the mutant allele approaches fixation (q=1). The biological meaning of calculating $\alpha^{*}$ also helps to understand how large effect of a mutant allele enhances its fixation in a mixed mating system, given the strength of selection and a mutation rate. Note that a mutant allele effect on fitness could be implied from the relationship between the allele effect and selection coefficient (Falconer and Mackay 1996). All fixed mutant alleles contribute to the genetic load at the population level. This analysis is analogous to searching for a critical migration rate beyond which a migrant allele swamps local resident alleles and tends to fixation (Hu 2011). In the following parts of this section, we show that different selfing rates are required to fix mutant alleles expressed in the gametophyte or the sporophyte phase only, or in two phases.

For genes expressed in the gametophyte phase only ($s_{\mathrm{hap}}\neq 0$, $s_{\mathrm{het}}=s_{\mathrm{hom}}=0$), the mutant allele frequency (*q*) could consistently increase until fixation as long as its frequency always increases (Δq>0). From equation (1), the critical selfing rate, denoted by $\alpha_{1}^{*}$, is derived by setting Δq=0 and substitution of q=1, i.e.


(2) $$\alpha_{1}^{*}=1-\frac{\mu }{s_{\mathrm{hap}}}{\left(1-\frac{\mu }{s_{\mathrm{hap}}}\right)}^{-1}\;$$

It can be shown that the partial differential $\partial \alpha_{1}^{*}/\partial s_{\mathrm{hap}}$ is greater than zero for the deleterious mutant allele ($s_{\mathrm{hap}}>0$), indicating that high selfing rates are required to fix a deleterious allele. The partial differential $\partial \alpha_{1}^{*}/\partial \mu$ is smaller than zero for the deleterious mutant allele, indicating that a lower selfing rate is required when the mutation rate is higher. Figure 1 shows that the critical selfing rate $\alpha_{1}^{*}$ increases as the strength of gametophytic selection becomes strong. Note that in the case of pollen (male gametes) selection only ($s_{P}\neq 0$, $s_{O}=0$) or the case of ovules (female gametes) selection only ($s_{P}=0$, $s_{O}\neq 0$), $\alpha_{1}^{*}$ can be calculated from equation (2). However, $\alpha_{1}^{*}$ remains unaltered if the composite selection coefficient $s_{\mathrm{hap}}$ is constant even if there is variation between male and female haploid fitnesses ($s_{O}\neq s_{P}\neq 0$) in the gametophyte phase.

For genes expressed in the sporophyte phase only ($s_{O}=s_{P}=0$, $s_{\mathrm{het}}\neq 0$, $s_{\mathrm{hom}}\neq 0$), the critical selfing rate, denoted by $\alpha_{2}^{*}$, is obtained from equation (1) by setting Δq=0 and substitution of q=1,


(3) $$\alpha_{2}^{*}\;=\;1-\frac{1}{2}\cdot \frac{\mu +s_{\mathrm{hom}}}{s_{\mathrm{het}}}{\left(1-\frac{1}{2}\cdot \frac{\mu +s_{\mathrm{hom}}}{s_{\mathrm{het}}}\right)}^{-1}$$

**Fig. 2.**
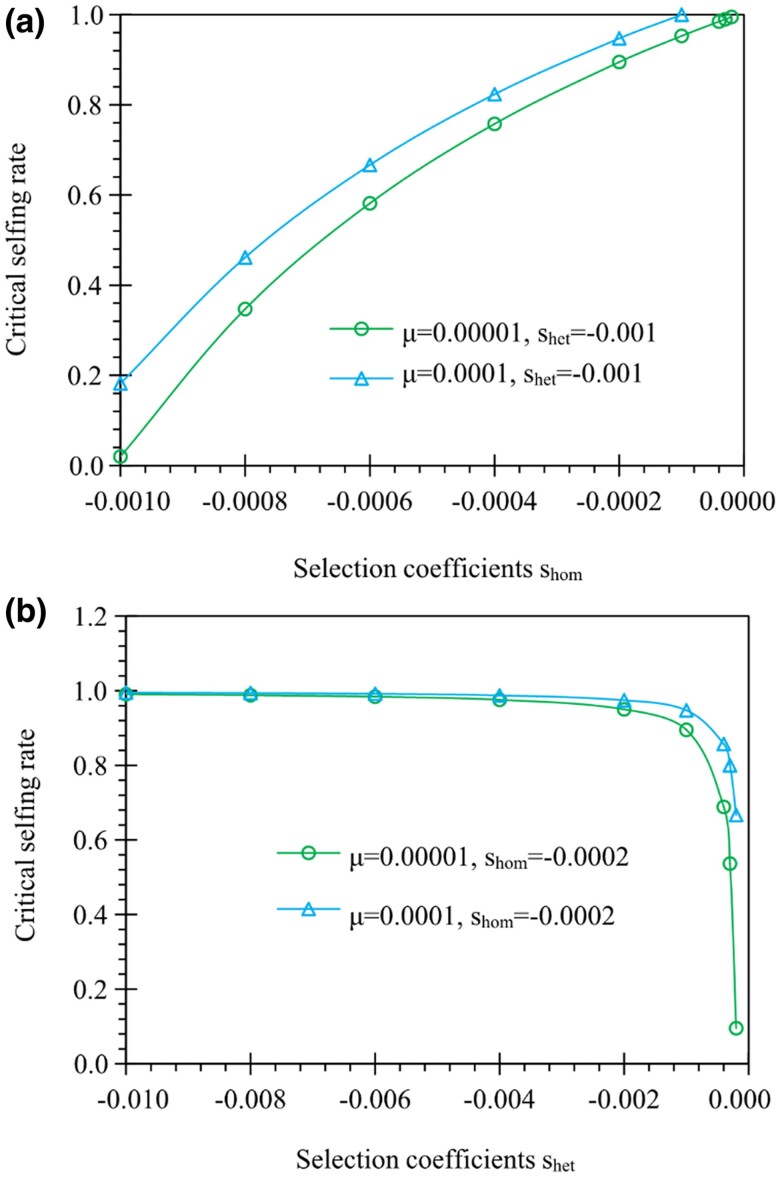
Examples of the critical selfing rate for genes expressed in the sporophyte phase only. Results are derived from equation (3). In a), the critical selfing rate increases with homozygotic selection ($s_{\mathrm{hom}}$) under a constant heterozygotic selection ($s_{\mathrm{het}}=-0.001$). In b), the critical selfing rate decreases with heterozygotic selection ($s_{\mathrm{het}}$) under a constant homozygotic selection ($s_{\mathrm{hom}}=-0.0002$). In a and b), the critical selfing rate has a positive relationship with the mutation rate ($\mu =10^{-5}$ and 10^−4^).

Equation (3) indicates that homozygotic ($s_{\mathrm{hom}}$) and heterozygotic selection ($s_{\mathrm{het}}$) oppositely affect the critical selfing rate $\alpha_{2}^{*}$. This is because selfing reduces heterozygosity but increases homozygosity. Given $0\leq \alpha_{2}^{*}\leq 1$, the condition of $0\leq (\mu +s_{\mathrm{hom}})/s_{\mathrm{het}}\leq 1$ is needed. For the deleterious mutant allele ($s_{\mathrm{hom}}<0$, $s_{\mathrm{het}}<0$), it can be shown that the relationships of $\partial \alpha_{2}^{*}/\partial s_{\mathrm{het}}<0\;$ and $\partial \alpha_{2}^{*}/\partial s_{\mathrm{hom}}>0$ hold. The critical selfing rate $\alpha_{2}^{*}$ increases as the homozygotic selection coefficient ($s_{\mathrm{hom}}$) increases (Fig. 2a) but decreases as the heterozygotic selection coefficient ($s_{\mathrm{het}}$) increases (Fig. 2b). The critical selfing rate $\alpha_{2}^{*}$ increases as the mutation rate increases ($\partial \alpha_{2}^{*}/\partial \mu >0\;$). For the advantageous mutant allele ($s_{\mathrm{hom}}>0$, $s_{\mathrm{het}}>0$), opposite patterns are expected for $\alpha_{2}^{*}$ in response to homozygotic and heterozygotic selection ($\partial \alpha_{2}^{*}/\partial s_{\mathrm{het}}>0\;$, $\partial \alpha_{2}^{*}/\partial s_{\mathrm{hom}}<0$). High mutation reduces the critical selfing rate $\alpha_{2}^{*}$ ($\partial \alpha_{2}^{*}/\partial \mu <0$).

For genes expressed in both gametophytic and sporophytic phases ($s_{O}\neq 0,s_{P}\neq 0$, $s_{\mathrm{het}}\neq 0$, $s_{hom}\neq 0$), the critical selfing rate, denoted by $\alpha_{3}^{*}$, is obtained from equation (1):


(4) $$\alpha_{3}^{*}=1-\frac{1}{2}\cdot \frac{\mu +s_{\mathrm{hom}}}{s_{\mathrm{het}}+s_{\mathrm{hap}}/2}{\left(1-\frac{1}{2}\cdot \frac{\mu +s_{\mathrm{hom}}}{s_{\mathrm{het}}+s_{\mathrm{hap}}/2}\right)}^{-1}$$

**Fig. 3.**
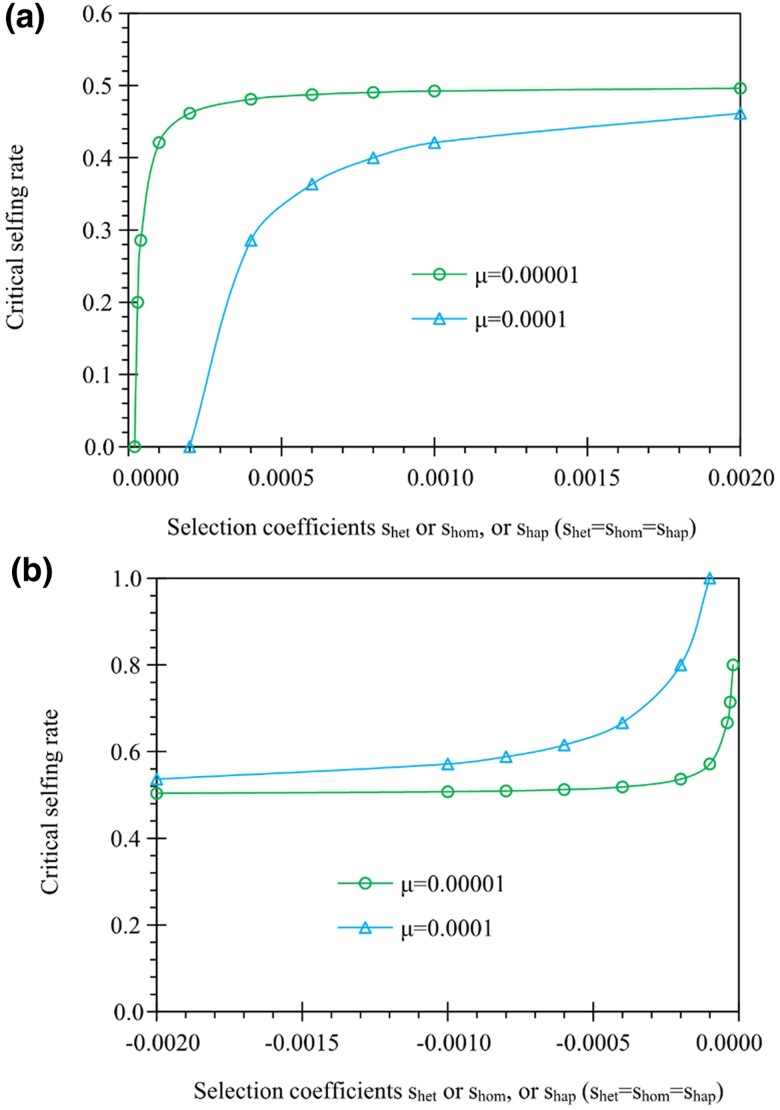
Examples of the critical selfing rate for genes expressed in both gametophyte and sporophyte phases. Results are derived from equation (4) under two mutation rates ($\mu =10^{-5}$ and 10^−4^). a) The critical selfing rate has a negative relationship with the mutation rate in antagonistic selection, b) but a positive relationship with the mutation rate in synergistic selection. In a), the critical selfing rate increases as the selection coefficients $s_{\mathrm{hap}}$, $s_{\mathrm{hom}}$, and $s_{\mathrm{het}}$ increase from 0.00002 to 0.002. In b), the critical selfing rate increases as the selection coefficients $s_{\mathrm{hap}}$, $s_{\mathrm{hom}}$, and $s_{\mathrm{het}}$ increase from −0.002 to −0.00002.

Given $0\leq \alpha_{3}^{*}\leq 1$, the condition of $0\leq (\mu +s_{\mathrm{hom}})/(s_{\mathrm{het}}+s_{\mathrm{hap}}/2)\leq 1$ is needed. When antagonistic selection occurs between gametophytic and sporophytic phases, allelic polymorphisms could likely be maintained even without mutation inputs. However, selfing could evolve the mutant allele toward fixation. For instance, in the case of negative gametophytic selection ($s_{\mathrm{hap}}>0$) combined with positive sporophytic selection ($s_{\mathrm{hom}}>0$ and $s_{\mathrm{het}}>0$), the critical selfing rate $\alpha_{3}^{*}$ increases as the selection coefficients ($s_{\mathrm{hap}}$, $s_{\mathrm{hom}}$, and $s_{\mathrm{het}}$) increase (Fig. 3a), but only a moderate or lower selfing rate $\alpha_{3}^{*}$ is required to fix the mutant allele. Higher mutation rates reduce the critical selfing rate $\alpha_{3}^{*}$. However, in the case of positive gametophytic selection but negative sporophytic selection ($s_{\mathrm{hap}}<0$, $s_{\mathrm{hom}}<0$, and $s_{\mathrm{het}}<0$), an opposite pattern is present, and the critical selfing rate $\alpha_{3}^{*}$ decreases as the absolute selection strengths increase. Also, an opposite pattern occurs regarding the mutation effects. A higher critical selfing rate $\alpha_{3}^{*}$ is yielded under higher mutation rates (Fig. 3b).

When synergistic selection between phases occurs, such as negative selection in both phases ($s_{\mathrm{hap}}>0$, $s_{\mathrm{hom}}<0$, and $s_{\mathrm{het}}<0$), only mutation counteracts the negative selection and maintains a low level of allelic polymorphisms. A high critical selfing rate $\alpha_{3}^{*}$ is needed to fix the deleterious mutant allele only when the condition of $s_{\mathrm{het}}+\frac{1}{2}s_{\mathrm{hap}}>\mu +s_{\mathrm{hom}}>0$ is met, which otherwise the mutant allele is inevitably extinct. Under this situation, the required mutation rate is greater than the selection coefficient of genotype *aa* ($\mu >|s_{\mathrm{hom}}$|) and the gametophytic selection is much stronger than the sporophytic selection ($s_{\mathrm{hap}}>2|s_{\mathrm{het}}|$). However, when the mutant allele is favorable in both phases ($s_{\mathrm{hap}}<0$, $s_{\mathrm{hom}}>0$, and $s_{\mathrm{het}}>0$), it approaches fixation in any mating system.

### Fixation Under Random Drift Process

In a finite population of effective population size $N_{e}$, both genetic drift and selfing facilitate fixation of the mutant allele. Here, we concentrate on Wright's method to derive the approximate fixation probability of a mutant allele under an equilibrium among irreversible mutation, genetic drift and selection effects (Wright 1931, 1938). This method is based on the density distribution of allele frequency rather than the diffusion model. We also apply Kimura's method to give the approximate fixation probability under an equilibrium between selection and genetic drift effects (Appendix B, Supplementary Material online; Kimura 1962). The results from these two methods are compared with numerical examples to illustrate how selfing shapes fixation of the mutant allele.

### Fixation Probability

As in Wright (1938), we consider a mutant allele under irreversible mutation, selection and genetic drift processes in a population that follows the life cycle mentioned in the general assumptions. Equation (1) provides the average systematic change of the mutant allele frequency per generation, $M_{\delta q}=\Delta q$. We now proceed to consider the distribution of the mutant allele frequency under steady flux of irreversible mutation. According to Wright (1938), the joint effects of these processes generate a steady density distribution of allele frequency under steady flux of mutation. All class frequencies ($q=\frac{1}{2N_{e}},\ldots ,1-\frac{1}{2N_{e}}$) would fall off at a constant rate (*K*) as genes irreversibly drift into fixation (Wright 1938, 1969). Under the condition of $2N_{e}\mu <1$, the decaying rate *K* generated by irreversible mutation is smaller than the decaying rate of a neutral allele ($1/2N_{e}$) under a solely drift process (Wright 1931, 1969). The irreversible mutation would ultimately go to fixation even if opposed by selection (Wright 1938). Here, we analyze how selfing modifies the rate of allele fixation (*K*) and the fixation probability of the mutant allele under flux equilibrium.

Let ϕ(q) be the density distribution of the mutant allele frequency (*q*). The variance of the distribution of allele frequency, generated by genetic drift, increases by $\sigma_{\delta q}^{2}=\frac{q(1-q)}{2N_{e}}$ per generation, where δq represents a random change of allele frequency. Following Wright (1938, 1942), two assumptions are postulated about the change in mean and variance of the mutant allele frequency due to irreversible mutation, selection, and genetic drift:


(5) $$\bar{q+\Delta q+\delta q}=\bar{q}+K(1-\bar{q})$$

(6) $$\sigma_{\left(q+\Delta q+\delta q\right)}^{2}=(1-K)\sigma_{q}^{2}+K(1-\bar{q}{)}^{2}$$

The second term on the right side of equation (5) represents the average change of the mutant allele frequency due to the irreversible mutation. Note that the mean of the random change δq of allele frequency is equal to zero. The right side of equation (6) consists of the variance generated by genetic drift and weighted by 1−K (the remaining proportion after fixation) and the variance generated by influx of the mutant allele. Equations (5) and (6) ensure the density distribution of allele frequency at flux equilibrium (Wright 1938, 1942).

To derive the density function of allele frequency ϕ(q) and the decaying rate *K*, the following relations are approximated (Wright 1937, 1942). Under influx equilibrium, the frequency of the allele frequency at subterminal class $q=\frac{1}{2N_{e}}$ is approximated by twice the frequency of new mutations, i.e.


(7) $$f\left(\frac{1}{2N_{e}}\right)=4N_{e}\mu f(0)$$

Where f(0) is the probability of the mutant allele frequency at class q=0. The frequency of the allele frequency at the subterminal class $q=1-\frac{1}{2N_{e}}$ is twice the flux (2*K*) through all heteroallelic classes (Wright 1938, 1969),


(8) $$K=\frac{1}{2}f\left(1-\frac{1}{2N_{e}}\right)\approx \frac{1}{4N_{e}}\phi \left(1-\frac{1}{2N_{e}}\right).$$

Following Wright's idea (Wright 1938), the derivation of the density function of allele frequency at nonterminal classes (q≠0 and 1), ϕ(q), is briefly summarized as follows. When the effective population size is small, the mutation effects on ϕ(q) are neglected in comparison with selection effects. From equation (1), the change of the mutant allele frequency, Δq, is approximated by,


(9) Δq=(S+Tq)q(1−q)

Where $S=-\frac{1}{2}(1-F_{is})s_{\mathrm{hap}}+(1-F_{is})s_{\mathrm{het}}+F_{is}s_{\mathrm{hom}}$ and $T=(1-F_{is})(s_{\mathrm{hom}}-2s_{\mathrm{het}})$. The coefficients of *S* and *T* remain in the same order as $s_{\mathrm{hap}}$, $s_{\mathrm{hom}}$, and $s_{\mathrm{het}}\;$ in magnitude. Equation (9) is equivalent to equation (17) of Wright (1938). Under ignorable effects of *K* on ϕ(q) at the nonterminal classes due to small mutation rate, a steady density distribution of gene frequency at any specific class can be attained under selection and drift effects, like the derivation of equation (19) of Wright (1938), which further yields ϕ(q), i.e. equation (27) of Wright (1938), after sophisticated algebraic calculations. The irreversible mutation is then considered from its effects on average change of gene frequency and on the allele frequencies at subterminal classes (equations [7 and 8]). The probabilities of allele frequencies at subterminal classes, $f\left(\frac{1}{2N_{e}}\right)$ and $f\left(1-\frac{1}{2N_{e}}\right)$, are also applied to derive ϕ(q) and *K* and finally yield their calculations, i.e. equations (35) and (37) of Wright (1938).

We now straightforward apply Wright's results to calculate ϕ(q) at flux equilibrium. Substitution of the selection coefficients *s* and *t* in equation (35) of Wright (1938) with *S* and *T* in equation (9), respectively, yields,


(10) $$\begin{array}{rl} \phi (q)\;= & \frac{4N_{e}\mu }{q(1-q)}(e^{4N_{e}Sq+2N_{e}Tq^{2}}\\ & -\;qe^{2N_{e}S(1+q)+2N_{e}T(1+q^{2})}\frac{\psi (2N_{e}Sq,2N_{e}Tq^{2})}{\psi (2N_{e}S,2N_{e}T)}) \end{array}$$

Details for calculating 2D infinite series ψ(x,y) in equation (10) are provided by Wright (1938, 1942).

Two specific cases for the infinite series are $\psi (x,0)=(e^{x}-e^{-x})/2x$ and $\psi (0,y)=1+\frac{1}{3!}y+\frac{7}{5!}y^{2}+\frac{27}{7!}y^{3}+\cdots$. Note that Wright (1945) used a more general differential equation to derive ϕ(q) that is the same as equation (10) under flux equilibrium. From Wright (1945, 1969), a general calculation for $\psi (2N_{e}Sq,2N_{e}Tq^{2})$ is given by $\psi (2N_{e}Sq,2N_{e}Tq^{2})=(e^{2N_{e}Sq+N_{e}Tq^{2}}/q)\int e^{-2N_{e}Sq-N_{e}Tq^{2}}dq$. In the neutral case (S=T=0), the density distribution of the mutant allele frequency reduces to $\phi (q)=\frac{4N_{e}\mu }{q}$, which is close to $4N_{e}\mu q^{4N_{e}\mu -1}$ under low mutation rates (Wright 1969). The decaying rate for all classes of neutral allele frequencies is K=μ (Wright 1931).

The frequency of allele frequency *q*, f(q), is approximated by $\frac{1}{2N_{e}}\phi (q)$. According to Wright (1938, 1969), the fixation probability of the mutant allele with an initial frequency $\frac{1}{2N_{e}}$ is approximated by $u\left(\frac{1}{2N_{e}}\right)=$  $f\left(1-\frac{1}{2N_{e}}\right)/f\left(\frac{1}{2N_{e}}\right)$, meaning the probability of fixation conditional on the occurrence of the mutant allele. By neglecting the terms with the second and higher orders of selection coefficients ($S^{2}$, $T^{2}$, $S/N_{e}$, and $T/N_{e}$), we obtain the fixation probability of the mutant allele in a mixed mating system,


(11) $$u\left(\frac{1}{2N_{e}}\right)=\frac{e^{2(2N_{e}-1)S+2(N_{e}-1)T}-\left(1-\frac{1}{2N_{e}}\right)\gamma_{1}e^{(4N_{e}-1)S+(2N_{e}-1)T}}{e^{2S}-\frac{1}{2N_{e}}\gamma_{2}e^{\left(2N_{e}+1)S+N_{e}T\right)}}\;$$

where $\gamma_{1}=\frac{\psi ((2N_{e}-1)S,2(N_{e}-1)T)}{\psi (2N_{e}S,2N_{e}T)}$ and $\gamma_{2}=\frac{\psi (S,\;T/2N_{e})}{\psi (2N_{e}S,2N_{e}T)}$ can be numerically calculated. From equation (11), we can assess how selfing regulates fixation probability of the mutant allele. Note that the density distribution of the mutant allele frequency is the function of the mutation rate (*μ*), but the fixation probability of the mutant allele is not due to the cancellation of the mutation rate between the numerator, $f\left(1-\frac{1}{2N_{e}}\right)$, and the denominator, $f\left(\frac{1}{2N_{e}}\right)$ in calculation.

Applying equation (37) of Wright (1938), we obtain the rate of fixation of genes (*K*) due to irreversibly drifting into the terminal class q=1 in a mixed mating system,


(12) $$\begin{array}{r} K=\mu \left(e^{4N_{e}S+2N_{e}T}-\frac{1}{\psi (2N_{e}S,2N_{e}T)}\left(2N_{e}S+\frac{4}{3}N_{e}T\right)e^{2N_{e}S+N_{e}T}\right) \end{array}$$

The rate of fixation (*K*) reduces to $\mu \left(1+2N_{e}S+\frac{2}{3}2N_{e}T\right)$ for small vales of $2N_{e}S$ and $2N_{e}T$. The mutant allele would ultimately lead to fixation even if it is opposed by negative selection (Wright 1938). The ratio K/μ is equal to 1 under the neutral process, but greater or smaller than 1 when the mutant allele is favorable or deleterious in two alternative generations, respectively. In the case of $s_{hom}=2s_{het}$ (a linear additive selection model in the sporophyte phase), we obtain T=0 and analytical $\psi (2N_{e}S,0)$. The ratio K/μ is derived as:


(13) $$\begin{array}{r} \frac{K}{\mu }=\frac{4N_{e}\left(-\frac{1}{2}(1-F_{is})s_{\mathrm{hap}}+(1+F_{is})s_{\mathrm{het}}\right)}{1-\exp\left(-4N_{e}\left(-\frac{1}{2}(1-F_{is})s_{\mathrm{hap}}+(1+F_{is})s_{\mathrm{het}}\right)\right)}, \end{array}$$

This indicates that selfing regulates the rate of fixation of genes (*K*).

In Appendix B, Supplementary Material online, we also provide the formulae for calculating the fixation probability of the mutant allele from the diffusion model (Kimura 1962). Partial selfing together with phase variation in selection in a life cycle can be assessed. This probability of fixation is derived under an equilibrium between selection and genetic drift effects, without the irreversible mutation process.

### Numerical Comparisons

Here, we assess the fixation probability of the mutant allele under different cases of gene expression in a single phase or in two phases. We also compare the predictions by Wright's and Kimura's methods and check their appropriateness through MC simulations. Supplementary table S1, Supplementary Material online summarizes the analytical comparisons derived from the two methods in several specific cases. Generally, there are slight differences between the results from the two methods for genes expressed in the gametophytic phase only ($s_{\mathrm{hap}}\neq 0,s_{\mathrm{hom}}=s_{\mathrm{het}}=0$), or for genes expressed in the sporophyte phase only ($s_{\mathrm{hap}}=0,s_{\mathrm{hom}}=2s_{\mathrm{het}}(\neq 0)$), and for genes expressed in two phases with additive selection model ($s_{\mathrm{hap}}\neq 0,s_{\mathrm{hom}}=2s_{\mathrm{het}}(\neq 0)$). In the case of antagonistic selection (nonadditive selection in sporophyte phase; $s_{\mathrm{hap}}\neq 0,s_{\mathrm{hom}}\neq 2s_{\mathrm{het}}(\neq 0)$), the following condition could be maintained under $-\frac{1}{2}\left(1-\frac{\alpha }{2-\alpha }\right)s_{\mathrm{hap}}+\left(1-\frac{\alpha }{2-\alpha }\right)s_{\mathrm{het}}+$  $\frac{\alpha }{2-\alpha }s_{\mathrm{hom}}=0\;$, which leads to S=0 but T≠0, and yields the selfing rate of $\alpha =1-\frac{s_{\mathrm{hom}}}{s_{\mathrm{hap}}-2s_{\mathrm{het}}+s_{\mathrm{hom}}}$. Calculations of ψ(0,y) and integral of an error function are needed.

**Fig. 4.**
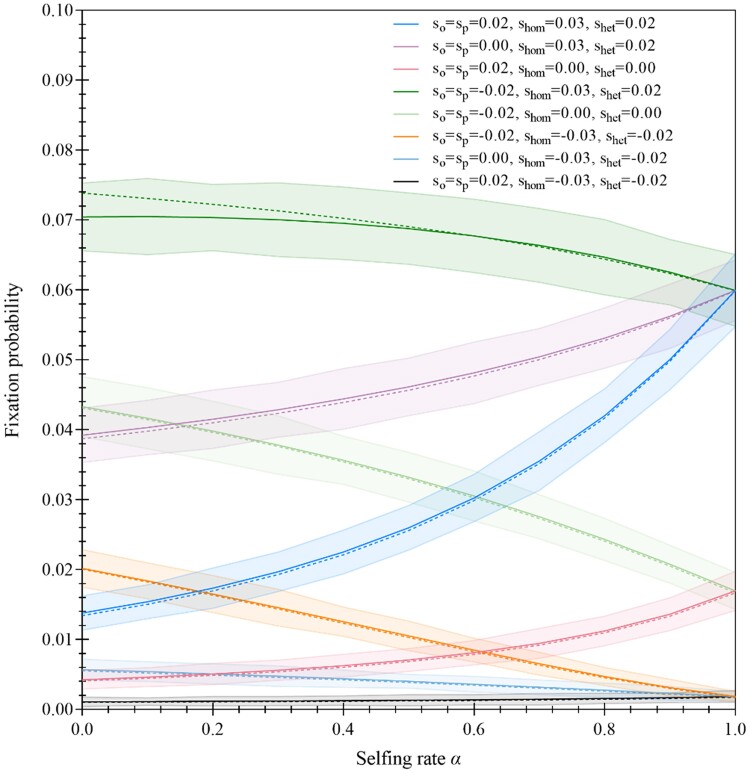
Examples of fixation probabilities under different cases of gametophytic and sporophytic selection. The solid lines in each case are the results obtained from equation (9), and the dashed lines are the results obtained from equation (B1) in Supplementary material online. The results from MC simulations are indicated. For each case, 10,000 replicates are simulated to estimate fixation probability. One hundred datasets for each case are simulated to estimate mean and standard deviation of the fixation probability. Ninety-five percent CIs are showed in different colors. The effective population size for each case is set as $N_{e}=30$. The simulated cases are the gametophytic selection only ($s_{O}=s_{P}=0.02$, $s_{\mathrm{hom}}=0.00$, $s_{\mathrm{het}}=0.00$ for the deleterious mutant allele; $s_{O}=s_{P}=-0.02$, $s_{\mathrm{hom}}=0.00$, $s_{\mathrm{het}}=0.00$ for the favorable mutant allele), the sporophytic selection only ($s_{O}=s_{P}=0.00$, $s_{\mathrm{hom}}=-0.03$, $s_{\mathrm{het}}=-0.02$ for the deleterious mutant allele; $s_{O}=s_{P}=0.00$, $s_{\mathrm{hom}}=0.03$, $s_{\mathrm{het}}=0.02$ for the favorable mutant allele), the antagonistic selection ($s_{O}=s_{P}=-0.02$, $s_{\mathrm{hom}}=-0.03$, $s_{\mathrm{het}}=-0.02$; $s_{O}=s_{P}=0.02$, $s_{\mathrm{hom}}=0.03$, $s_{\mathrm{het}}=0.02$), and the synergistic selection ($s_{O}=s_{P}=0.02$, $s_{\mathrm{hom}}=-0.03$, $s_{\mathrm{het}}=-0.02$ for the deleterious mutant allele; $s_{O}=s_{P}=-0.02$, $s_{\mathrm{hom}}=0.03$, $s_{\mathrm{het}}=0.02$ for the favorable mutant allele).

Examples show that the fixation probabilities predicted by Wright's method are slightly greater than those predicted by Kimura's method (Fig. 4). Note that a Mathematica notebook using NIntegrate [] function is applied to calculate an integral part of equation (B1) in Appendix B, Supplementary Material online (Wolfram 1996). The effects of selfing on fixation of the mutant allele vary with phase variation in selection. Under the gametophytic selection only ($s_{\mathrm{hom}}=s_{\mathrm{het}}=0$), selfing facilitates fixation of the deleterious mutant allele (e.g. $s_{O}=s_{P}=0.02\;$), but the fixation probability is smaller than the genetic drift rate, ½ $N_{e}=1/60$, except for the cases with high selfing rates. However, selfing impedes fixation of the favorable mutant allele ($s_{O}=s_{P}=-0.02$). This is because selfing reduces efficacy of selection in the gametophytic phase.

Under the sporophytic selection only ($s_{O}=s_{P}=0$), selfing facilitates fixation of the favorable mutant allele (e.g. $\;s_{\mathrm{hom}}=0.03,s_{\mathrm{het}}=0.02$), but impedes fixation of the deleterious mutant allele ($\;s_{\mathrm{hom}}=-0.03,s_{\mathrm{het}}=-0.02$). This is because selfing increases homozygosity, which indirectly facilitates or impedes efficacy of selection in the sporophytic phase. The fixation probabilities in the latter case ($\;s_{\mathrm{hom}}=-0.03,s_{\mathrm{het}}=-0.02$) are smaller than the fixation probability of a neutral allele even under high selfing rates.

Under both gametophytic and sporophytic selection, the fixation probability of the mutant allele is related to the type of biphasic selection. Under antagonistic selection between alternative generations, selfing facilitates fixation of the mutant allele when it is deleterious in the gametophytic phase but favorable in the sporophyte phase ($s_{O}=s_{P}=0.02,\;s_{\mathrm{hom}}=0.03,s_{\mathrm{het}}=0.02$). An opposite pattern occurs when the mutant allele is favorable in the gametophytic phase but deleterious in the sporophyte phase ($s_{O}=s_{P}=-0.02,\;s_{\mathrm{hom}}=-0.03,s_{\mathrm{het}}=-0.02$).

Similarly, under synergistic selection between alternative generations, selfing slightly increases the fixation probability of the mutant allele when it is deleterious in both phases ($s_{O}=s_{P}=0.02,\;s_{\mathrm{hom}}=-0.03,s_{\mathrm{het}}=-0.02$), but the fixation probability is less than $1/2N_{e}$. However, selfing impedes fixation of the mutant allele when it is favorable in both phases ($s_{O}=s_{P}=-0.02,\;s_{\mathrm{hom}}=0.03,s_{\mathrm{het}}=0.02$). In general, selfing exerts both direct (gametophytic phase) and indirect (sporophytic phase) effects on regulating fixation of the mutant allele when the mutant allele is pleiotropic in two phases.

To check theoretical predictions, MC simulations are conducted, and the scripts in C for simulations are provided in Supplementary material online. A generally good agreement between theoretical predictions and simulation results is present in all cases (Fig. 4). All theoretical predictions are within 95% confidence interval (CI) of the simulation distributions.

**Fig. 5.**
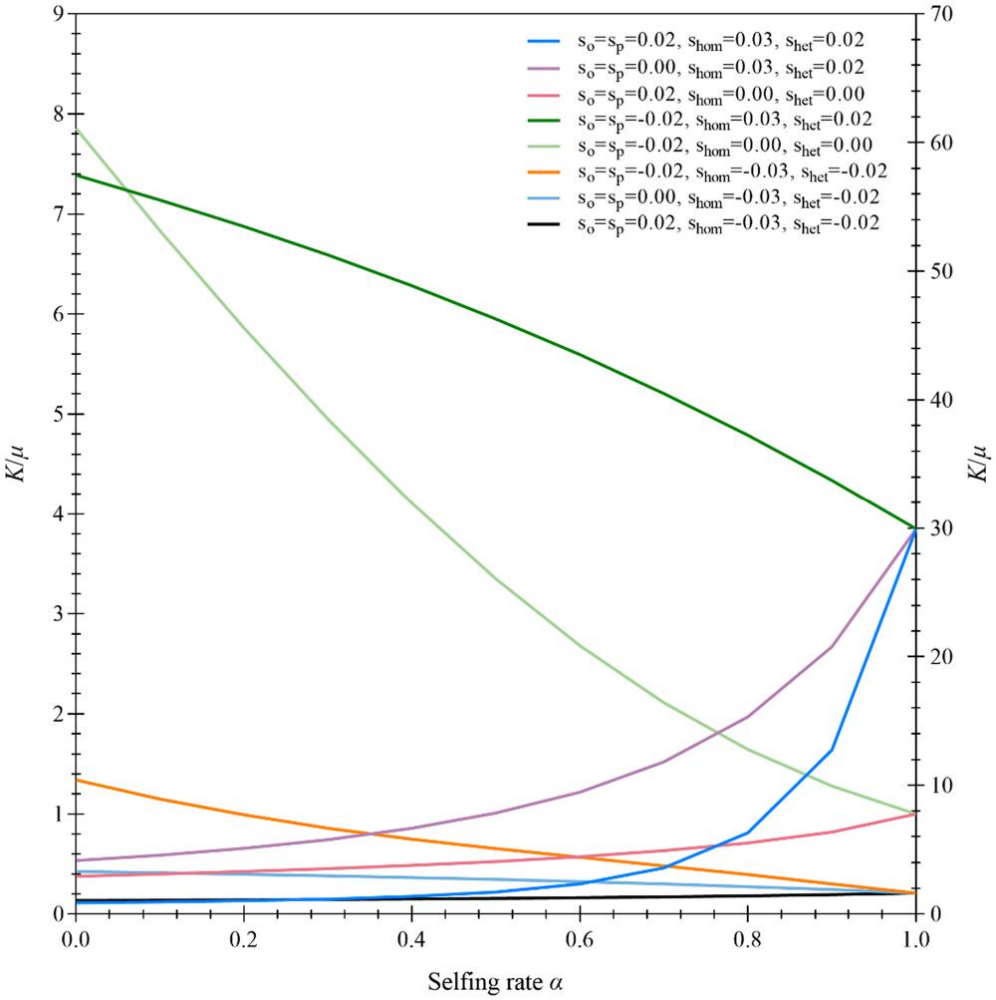
Examples of the K/μ ratio under different cases of gametophytic and sporophytic selection. The results are obtained from equation (10). Selection coefficients in each case are indicated in the figure, with a commonly used parameter of the effective population size $N_{e}=30$. The cases with the first *y*-axis are the gametophytic selection only ($s_{O}=s_{P}=0.02$, $s_{\mathrm{hom}}=0.00$, $s_{\mathrm{het}}=0.00$ for the deleterious mutant allele; $s_{O}=s_{P}=-0.02$, $s_{\mathrm{hom}}=0.00$, $s_{\mathrm{het}}=0.00$ for the favorable mutant allele), the sporophytic selection against the deleterious mutant allele ($s_{O}=s_{P}=0.00$, $s_{\mathrm{hom}}=-0.03$, $s_{\mathrm{het}}=-0.02$), the antagonistic selection ($s_{O}=s_{P}=-0.02$, $s_{\mathrm{hom}}=-0.03$, $s_{het}=-0.02$), and the synergistic selection against the deleterious mutant allele ($s_{O}=s_{P}=0.02$, $s_{\mathrm{hom}}=-0.03$, $s_{\mathrm{het}}=-0.02$). The cases with the second y-axis are the antagonistic selection ($s_{O}=s_{P}=0.02$, $s_{\mathrm{hom}}=0.03$, $s_{\mathrm{het}}=0.02$), the sporophytic selection for the favorable mutant allele ($s_{O}=s_{P}=0.00$, $s_{\mathrm{hom}}=0.03$, $s_{\mathrm{het}}=0.02$), and the synergistic selection for the favorable mutant allele ($s_{O}=s_{P}=-0.02$, $s_{\mathrm{hom}}=0.03$, $s_{\mathrm{het}}=0.02$).

It is interesting to look at how selfing affects the rate of fixation of genes relative to the mutation rate (*K/μ*) derived from equation (12). Figure 5 shows that the ratio *K/μ* exhibits opposite patterns to those of the fixation probabilities (Fig. 4) in different cases of selection in the gametophytic and sporophytic phases. The role of selfing is different in changing *K/μ* and the fixation probability of the mutant allele (Figs. 4 and 5).

To assess genetic drift effects, we calculate fixation probability based on equation (9) and use the same settings of selection coefficients as in Fig. 4 except for a smaller effective population size (supplementary fig. S1, Supplementary Material online). Compared with Fig. 4, the fixation probability of the mutant allele increases in each case when the genetic drift effect is large. However, compared with Fig. 5, the ratio K/μ exhibits different patterns under a smaller effective population size ($N_{e}=15$ in supplementary fig. S2, Supplementary Material online). The ratio K/μ decreases as the genetic drift effect increases and so does the rate of fixation of genes (*K*). The joint effects of selfing and genetic drift improve fixation of the mutant allele.

## Discussion

Alternation of generations (gametophyte and sporophyte phases) in a life cycle has multiple effects during the evolution of many multicellular eukaryotes. This includes less accumulation of deleterious alleles compared with asexual species (e.g. Muller's ratchet process) and the recombination for generating new genotypes for adaptation to diverse habitats. With alternative generations in a life cycle, deleterious mutant alleles can be masked from selection in heterozygotes in the sporophyte phase but are effectively purged in the gametophyte phase (Cervantes et al. 2023). This masking effect results in maintenance of some recessive alleles that are expressed in the sporophyte phase only, producing the mutation load in the sporophyte phase that is expected to be twice that in the gametophyte phase. The advantage of alternative generations lies in copying with diverse environments occurring in the gametophyte phase versus in the sporophyte phase (Barton et al. 2007). Here, we show that selfing can regulate both the purging effects in the gametophyte phase and the masking effect in the sporophyte phase. Selfing has the function similar to the asexual reproduction to accumulate deleterious alleles, and this is rarely emphasized in the literature.

Previous studies have addressed the effects of selfing on population evolution (Wright 1969), including the joint effects of selfing with background selection (Glémin and Ronfort 2013; Kamran-Disfani and Agrawal 2014), the joint effects of selfing with recombination (Sianta et al. 2023), the relative effects of selfing versus genetic drift (Charlesworth 1992; Glemin 2003), the effects of selfing on accumulating deleterious mutations (Arunkumar et al. 2013; Xu 2022), and the effects of selfing on speciation (Marie-Orleach et al. 2022). This study examines additional joint effects where selfing interacts with phase variation in selection in plant life cycle to shape the fixation of mutant alleles, which has not been fully explored in theory (Hu 2015; Li et al. 2023a, 2023b).

It is worth mentioning that based on the diffusion model (Kimura 1962), the fixation probability of a mutant allele expressed in the sporophyte phase only is independent of selfing rate under a liner additive selection model (Caballero and Hill 1992). This can also be verified from Appendix B, Supplementary Material online when $s_{\mathrm{hom}}\;$ is equal to $2s_{\mathrm{het}}$ for the mutant allele with an initial frequency of 1/2N (*N* is the actual population size and $N_{e}=N/(1+F_{is})$). If the initial allele frequency is $1/2N_{e}$, the fixation probability $u\left(\frac{1}{2N_{e}}\right)$ is related to the selfing rate (supplementary table S1, Supplementary Material online) and the relationship of $u\left(\frac{1}{2N_{e}}\right)=(1+F_{is})u\left(\frac{1}{2N}\right)$ holds. Also, the fixation probability is related to the selfing rate in the presence of dominant selection in the sporophyte phase only. For deleterious alleles expressed in the gametophyte phase only, both $u\left(\frac{1}{2N_{e}}\right)$ and $u\left(\frac{1}{2N}\right)$ increase as the selfing rate increases but a reverse relationship of $u\left(\frac{1}{2N}\right)=(1-F_{is})u\left(\frac{1}{2N_{e}}\right)$ holds. These two fixation probabilities are equal under random mating (*F_is_* = 0).

Two conclusions can be drawn about the role of selfing in shaping fixation of a mutant allele: (i) in an infinitely large population with partial selfing, the critical selfing rate varies with species in fixing deleterious alleles. Partial selfing species could harbor different amounts of deleterious alleles when their critical selfing rates differ in fixing mutant alleles expressed in the gametophyte or the sporophyte phase only, or in both phases. (ii) In a finite population with partial self-fertilization, selfing facilitates fixation of the mutant allele when it is deleterious in the gametophyte phase or when it is only expressed and favorable in the sporophyte phase under nonlinear additive selection model. Selfing impedes fixation of the mutant allele when it is favorable in the gametophyte phase or when it is only expressed and deleterious in the sporophytic phase. Effects of selfing on fixing pleiotropic genes depend on the relative strengths between two phases (Fig. 4).

Several cautions deserve attention about the theoretical assumptions. One is that the present theory only deals with a single locus with two alleles. When multiple loci are considered, the linkage disequilibrium (LD) between loci can be generated by partial selfing. Selfing reduces the recombination rate between linked loci (Nordborg 2000; Roze and Lenormand 2005), and hence the fixation probability of a mutant allele at one locus could potentially be affected by its linked locus. The analytical fixation probability has not been explored under LD between selective loci. When a neutral locus is linked with a selective locus, the fixation probability of its mutant neutral allele is not affected by its linked allele whether it is favorable (e.g. genetic hitchhiking effects [Maynard-Smith and Haig 1974]) or deleterious (e.g. background selection (Charlesworth et al. 1993), Birky and Walsh 1988). This is because LD between neutral and selective loci is transient and decays toward zero through recombination and genetic drift (Hill and Robertson 1966). The effective population size at a neutral locus transiently decreases by LD between the neutral and linked selective loci generated by selfing (Barton 2000) and hence does not alter the ultimate fixation probability of a neutral allele.

The theory considers a constant mutation rate. One caution is that selfing or inbreeding could reduce the point mutation rate or the mutation generated by recombination (Glemin et al. 2019; Li 2023a). The critical selfing rate in a large population could be modified for genes with various mutation rates. The mutation loads are also different when the mutation rates are unequal between species with different selfing rates (Nakayama et al. 2012). In a finite population, given the lower order of mutation rate, the fixation of a mutant allele is mainly governed by selection and genetic drift processes. The impact of this assumption is likely not serious in shaping fixation of a mutant allele in a finite population. In addition, in the presence of recurrent mutations, an equilibrium distribution of allele frequency ϕ(q) can be attained under the joint effects of recurrent mutation, selection, and genetic drift. In this situation, the rate of fixation (*K*) is expected to be zero (Wright 1931) and so is the fixation probability $u\left(\frac{1}{2N_{e}}\right)$.

Apart from the preceding assumptions, several implications can be obtained from the present theory. The first implication is concerned with deleterious mutations fixed by selfing and the assignment of genetic loads to the gametophyte phase versus the sporophyte phase at the population level. The overall population fitness can be calculated by multiplying the average fitness from the gametophyte ($\bar{w}$) and sporophyte ($\bar{W}$) phases (see Supplementary material online for calculating $\bar{w}$ and $\bar{W}$). The average population genetic load in the infinitely large population, denoted by $L(\;=\bar{w}\bar{W}-1)$, is calculated by:


$$L=q\left(-s_{\mathrm{hap}}+2p\left(1-\frac{\alpha }{2-\alpha }\right)s_{\mathrm{het}}+\left(q+\frac{\alpha }{2-\alpha }p\right)s_{\mathrm{hom}}\right)$$

which is equal to $-s_{\mathrm{hap}}+s_{\mathrm{hom}}$ when the deleterious allele is fixed (q=1). The loads due to fixation of the mutant allele are $-s_{\mathrm{hap}}$ and $s_{\mathrm{hom}}$ from the gametophyte and sporophyte phases, respectively. The average population genetic load in the finite population, denoted by $\bar{L}$, is calculated by:


$$\begin{array}{rl} \bar{L}\;= & \overset{1}{\underset{0}{\int }}\;q(-s_{\mathrm{hap}}+2p\left(1-\frac{\alpha }{2-\alpha }\right)s_{\mathrm{het}}\\ & +\;\left(q+\frac{\alpha }{2-\alpha }p\right)s_{\mathrm{hom}})\phi (q)dq \end{array}$$

which is a function of the selfing rate and selection coefficients. The genetic load also comes from both the gametophyte and sporophyte phases. The issue is how selfing assigns mutation loads to alternative phases in a life cycle. This is of biological significance since advantageous mutations are rare in natural populations (Eyre-Walker and Keightley 2007).

For genes expressed in the gametophyte phase only, the genetic load comes from the gametophytic phase arising from fixing deleterious mutations. Selfing increases genetic load and reduces population adaptation. For genes expressed in the sporophytic phase only, selfing impedes fixation of deleterious alleles and hence reduces genetic load. For genes expressed in both phases, selfing facilitates or impedes fixation of deleterious alleles, depending upon the mode of antagonistic selection, and the overall genetic load can increase or decrease. Thus, it is important to disentangle the relative contributions to the overall population genetic load among genes expressed in gametophyte phase only, in sporophyte phase only, and in both phases. This is likely related to the formation of alternative generations in a life cycle where relative lengths of gametophyte and sporophyte phases are different among species to reduce the overall genetic load. Previous theory indicates that selfing favors the gametophyte generation, while outcrossing favors the sporophyte generation (Otto and Marks 1996). A mixed mating system helps to form an optimal adaptive strategy for a species. Partial selfing regulates the relative proportions of mutation loads to each phase, which would vary among species with different selfing rates.

The second implication is concerned with the relative allele substitution rates among genes expressed in the gametophyte or the sporophyte phase only or in both phases. Selfing reduces the efficacy of purging deleterious alleles expressed in the gametophyte phase only or impedes positive selection of a favorable allele expressed in the gametophyte phase only. This reduces the evolutionary rates of gametophyte-specific genes. To the contrary, selfing facilitates positive selection of a favorable allele or enhances purging deleterious alleles expressed in the sporophyte phase only. This speeds up the evolutionary rates of sporophyte-specific genes. For genes under antagonistic selection between phases, selfing may facilitate or reduce the evolutionary rates of genes, depending on the relative strengths of selection in two phases. For genes with synergistic effects between phases, selfing impedes the substitution rates of either deleterious or favorable mutant alleles. This is essentially consistent with the patterns in terms of $K_{a}/K_{s}$ (the ratio of nonsynonymous substitutions/synonymous substitutions per nucleotide site; Li et al. 2023b).

The evolutionary rate of a gene can also be implied from the pattern of K/μ (equation [13]). Positive selection is indicated when K/μ>1, implying a high substitution rate. Purifying selection is indicated when K/μ<1, implying a low substitution rate. Neutral evolution is indicated when K/μ=1. The ratio K/μ could be estimated according to equation (13) when selection coefficients and effective population size are available in a mixed mating system.

The third implication is concerned with transcriptomic analysis of genes expressed in the gametophytic and sporophytic phases in a mixed mating system. Selfing regulates fixation probabilities of gametophyte- and sporophyte-specific genes and hence alters their expression evolution. Current analysis of gene expression does not distinguish the relative transcriptions from different alleles at a locus, especially for the samples collected from the sporophyte phase (Menzel et al. 2015; Frazee et al. 2021). Although gene expression is applied to inferring the underlying evolutionary processes (Bedford and Hartl 2009; Price et al. 2022; Zhang et al. 2022; Kagan and Hejnol 2024), polymorphic alleles at a locus are often mixed in their expression (the number of mRNA). The present study implicates the necessity of separate sampling tissues from the gametophyte phase (e.g. pollen) or from the sporophyte phase (e.g. plant leaves). Selfing facilitates fixation of deleterious alleles expressed in the gametophyte phase only and hence enhances polymorphisms, which also implicates the need of a certain sample size in empirical studies. A completely self-fertilized species leads to the same substitution rates among genes expressed in either the gametophytic phase or the sporophytic phase only or in both phases (Fig. 4). Also, the transcriptomes of the samples collected in the sporophyte phase are compound, including genes expressed in both gametophyte and sporophyte phases. Gene expression data from such samples cannot tell the pleiotropic genes from the genes expressed in a single phase only. Moreover, balancing selection for genes expressed only in sporophyte phase could generate similar patterns of gene expression to the antagonistic selection between two phases (Gilad et al. 2006). Therefore, to elucidate evolutionary processes underlying gene expression patterns, a deliberate experiment design is needed to collect samples from the gametophyte phase or from the sporophyte phase only.

## Supplementary Material

evae261_Supplementary_Data

## Data Availability

The Supplementary material includes Appendices A, B, C, and D, table S1, and figures S1 and S2. One Mathematica notebook for calculating fixation probability is provided in a separate document.
