# Supplementary material for: Selfing Shapes Fixation of a Mutant Allele Under Flux Equilibrium
Source: Genome Biol Evol. 2024 Dec 4;16(12):evae261. doi: 10.1093/gbe/evae261 (PMC11652729; doi:10.1093/gbe/evae261)
Supplement: evae261_Supplementary_Data [file evae261_supplementary_data.zip › Supplmentarymaterial20241024.docx]

**Supplementary Material**

**Selfing shapes fixation of a mutant allele under flux equilibrium**

Yu Xiao^1,2^, Yan-Wen Lv ^1,2^, Zi-Yun Wang^1,2^, Chao Wu^1,2^, Zi-Han He ^1,2^, Xin-Sheng Hu^1,2*^

1. College of Forestry and Landscape Architecture, South China Agricultural University, Guangzhou 510642, China

2. Guangdong Key Laboratory for Innovative Development and Utilization of Forest Plant Germplasm, Guangzhou 510642, China

Correspondence: Xin-Sheng Hu, Email: xinsheng@scau.edu.cn

**Appendix A: Systematic change of a mutant allele frequency**

The derivation is based on the life cycle mentioned in the main text. Consider one nuclear locus with two alleles (*A*, *a*) in a population. Allele *A* is the ancestral type and *a* is the mutant allele. Let $F_{is}$ be the inbreeding coefficient in the population. The genotypic frequencies in current adults at generation $t$ are expressed as $p_{AA}=p^{2}+pqF_{is}$, $p_{Aa}=2pq(1-F_{is})$, and $p_{aa}=q^{2}+pqF_{is}$ where $p$ and $q$ are the frequencies ($p+q$=1) of alleles *A* and *a*, respectively. Consider the selfing part, with a probability of α. Gametic selection works only on the heterozygotes. Let gametic fitness be 1 for allele *A* in pollen and ovules, 1-$s_{P}$ and 1-$s_{o}$ be the fitness of allele *a* in pollen and ovules, respectively. Distinct competition abilities are allowed between pollen and ovules when selection coefficients $s_{P}$ and $s_{O}$ are unequal. We may obtain that the average fitness is 1, 1-$\frac{1}{2}s_{o}$, and 1-$s_{o}$ for genotypes *AA*, *Aa*, and *aa* in ovules. The average fitness is 1, 1-$\frac{1}{2}s_{P}$, and 1-$s_{P}$ for genotypes *AA*, *Aa*, and *aa* in pollen. After gametic selection and selfing, the genotypic frequencies from the selfing part are summarized as follows:

$p_{AA.S}=p^{2}+\frac{1}{2}pq\left( 1+\frac{1}{2}\left( s_{o}+s_{P} \right) \right)+\frac{1}{2}pqF_{is}\left( 1-\frac{1}{2}\left( s_{o}+s_{P} \right) \right)$ (A1)

$p_{Aa.S}=pq(1-F_{is})$ (A2)

$p_{aa.S}=q^{2}+\frac{1}{2}pq\left( 1-\frac{1}{2}\left( s_{o}+s_{P} \right) \right)+\frac{1}{2}pqF_{is}\left( 1+\frac{1}{2}\left( s_{o}+s_{P} \right) \right)$ (A3)

Consider the outcrossing part, with a probability of 1-$\alpha$. The average fitness is $1-s_{o}q$ in ovules and $1-s_{P}q$ in pollen. After gametic selection and random combinations between pollen and ovules, the genotypic frequencies are:

$p_{AA.R}=p^{2}\left( 1+q\left( s_{o}+s_{P} \right) \right)$ (A4)

$p_{Aa.R}=2pq\left( 1+\frac{1}{2}\left( q-p \right)\left( s_{o}+s_{P} \right) \right)$ (A5)

$p_{aa.R}=q^{2}\left( 1-p\left( s_{o}+s_{P} \right) \right)$. (A6)

The average fitness in the gametophytic phase, denoted by $\bar{w}$, is derived as

$\bar{w}=\left( 1-\alpha\right)\left( 1-s_{O}q \right)\left( 1-s_{P}q \right)+\alpha\left( p_{AA}\cdot1+p_{Aa}\left( 1-\frac{1}{2}s_{O} \right)\left( 1-\frac{1}{2}s_{P} \right)+p_{aa}\left( 1-s_{O} \right)\left( 1-s_{P} \right) \right)$

$=1-\left( s_{O}+s_{P} \right)q$ (A7)

Consider a constant selfing rate for each genotype. Let $s_{hap}=s_{o}+s_{p}$. Combining the selfing and outcrossing parts yields the genotypic frequencies in a mixed mating system:

$p_{AA}^{*}=\alpha p_{AA.S}+\left( 1-\alpha\right)p_{AA.R}$

=$p^{2}+\frac{1}{2}\alpha pq\left( 1+F_{is} \right)+spq\left( \frac{1}{4}\alpha(1-F_{is})+\left( 1-\alpha\right)p \right)$ (A8)

$p_{Aa}^{*}=\alpha p_{Aa.S}+\left( 1-\alpha\right)p_{Aa.R}$

=$2pq\left( 1+\frac{1}{2}\alpha\left( 1+F_{is} \right) \right)+(1-\alpha)pq(q-p)s$ (A9)

$p_{aa}^{*}=\alpha p_{aa.S}+\left( 1-\alpha\right)p_{aa.R}$

= $q^{2}+\frac{1}{2}\alpha pq\left( 1+F_{is} \right)+spq\left( -\frac{1}{4}\alpha\left( 1-F_{is} \right)-\left( 1-\alpha\right)q \right)$ (A10)

Let $\mu$ be the mutation rate from allele *A* to *a*, and the backward mutation is neglected. The three genotypic frequencies become

$p_{AA}^{**}=\left( 1-\mu\right)^{2}p_{AA}^{*}$ (A11)

$p_{Aa}^{**}=2\mu\left( 1-\mu\right)p_{AA}^{*}+(1-\mu)p_{Aa}^{*}$ (A12)

$p_{aa}^{**}=\mu^{2}p_{AA}^{*}+\mu p_{Aa}^{*}+p_{aa}^{*}$ (A13)

Consider selection in the sporophyte phase. Let the fitness of three genotypes be $W_{AA}=1$ , $W_{Aa}=1+s_{het}$, and $W_{aa}=1+s_{hom}$. The average fitness in the sporophyte phase, denoted by $\bar{W}$, is calculated by $\bar{W}=W_{AA}p_{AA}^{***}+W_{Aa}p_{Aa}^{***}+W_{aa}p_{aa}^{***}$, and simplified as

$\bar{W}=1+2pq\left( 1-\frac{1}{2}\alpha(1+F_{is}) \right)s_{het}+q\left( q+\frac{1}{2}p\alpha\left( 1+F_{is} \right) \right)s_{hom}$ (A14)

After sporophytic selection, the genotypic frequencies can be calculated by $p_{AA}^{***}={w_{AA}p_{AA}^{**}}/{\bar{W},}$ $p_{Aa}^{***}=w_{Aa}p_{Aa}^{**}/\bar{W}$, and $p_{aa}^{***}=w_{aa}p_{aa}^{**}/\bar{W}$:

$p_{AA}^{***}=p\left( p+\frac{1}{2}\alpha q(1+F_{is}) \right)-2p\left( p+\frac{1}{2}\alpha q\left( 1+F_{is} \right) \right)\mu+pq\left( \left( 1-\alpha\right)p+\frac{1}{4}\alpha(1-F_{is}) \right)s_{hap}$

$-\left( 2pq\left( 1-\frac{1}{2}\alpha(1+F_{is}) \right)s_{het}+\left( q^{2}+\frac{1}{2}\alpha pq\left( 1+F_{is} \right)s_{hom} \right) \right)\left( p^{2}+\frac{1}{2}\alpha pq(1+F_{is}) \right)$

(A15)

$p_{Aa}^{***}=2pq\left( 1-\frac{1}{2}\alpha(1+F_{is}) \right)+2p\left( p-q+q\alpha(1+F_{is}) \right)\mu+\left( 1-\alpha\right)pq\left( q-p \right)s_{hap}$

$+\left( \left( 1-2pq\left( 1-\frac{1}{2}\alpha(1+F_{is}) \right) \right)s_{het}-\left( q^{2}+\frac{1}{2}\alpha pq(1+F_{is}) \right)s_{hom} \right)\left( 2pq\left( 1-\frac{1}{2}\alpha(1+F_{is}) \right) \right)$

(A16)

$p_{aa}^{***}=q^{2}+\frac{1}{2}\alpha pq\left( 1+F_{is} \right)+2pq\left( 1-\frac{1}{2}\alpha(1+F_{is}) \right)\mu-pq\left( \frac{1}{4}\alpha\left( 1-F_{is} \right)+\left( 1-\alpha\right)q \right)s_{hap}$

$+\left( -2pq\left( 1-\frac{1}{2}\alpha\left( 1+F_{is} \right) \right)s_{het}+\left( 1-\left( q^{2}+\frac{1}{2}\alpha pq(1+F_{is}) \right) \right)s_{hom} \right)\left( q^{2}+\frac{1}{2}\alpha pq(1+F_{is}) \right)$

(A17)

After algebraic calculations using above equations, the mutant allele frequency in the next adults before genetic drift is derived as

$q^{'}$=$p_{aa}^{***}+\frac{1}{2}p_{Aa}^{***}$

=$q+p\mu-\frac{1}{2}pq\left( 1-\frac{1}{2}\alpha\left( 1+F_{is} \right) \right)s_{hap}-pq\left( q-p \right)\left( 1-\frac{1}{2}\alpha\left( 1+F_{is} \right) \right)s_{het}+pq\left( q+\frac{1}{2}\alpha(1+F_{is}) \right)s_{hom}$

(A18)

Thus, the systematic change of the mutant allele frequency is then calculated as $\Delta q=q^{'}-q$.

**Appendix B: Fixation probability calculated by Kimura’s method**

Assume that the mutant allele goes to be fixed or lost before the occurrence of a next mutation. Selection and genetic drift ae the main processes to determine the trajectory of the mutant allele frequency. The fitnesses for alleles in the gametophyte phase and for genotypes in the sporophyte phase are set as those described in Appendix A. Kimura (1962) derived a stationary distribution of allele frequency and the fixation probability under an equilibrium of selection and genetic drift. We directly apply this result to a finite plant population with phase variation in selection during a life cycle. The fixation probability of the mutant allele with an initial frequency $q_{0}$ is calculated by

$u\left( q_{0} \right)=\frac{\int_{0}^{q_{0}} G(q)dq}{\int_{0}^{1} G(q)dq}$ (B1)

where

$G\left( q \right)=\exp\left( -\int\frac{2M_{\delta q}}{V_{\delta q}}dq \right)$ (B2)

in which $M_{\delta q}$ is the mean of the change in allele frequency per generation ($M_{\delta q}=\Delta q$), and $V_{\delta q}$ is $V_{\delta q}=\frac{q(1-q)}{2N_{e}}$. $G\left( q \right)$ is then derived as $G\left( q \right)=\exp\left( -4N_{e}\left( S+\frac{1}{2}Tq \right)q \right)$ by substituting $M_{\delta q}$ in equation (7) in the main text and $V_{\delta q}$ into equation (B2), where the capital *S* and *T* are $S=-\frac{1}{2}\left( 1-F_{is} \right)s_{hap}+\left( 1-F_{is} \right)s_{het}+{F_{is}s}_{hom}$ and $T=\left( 1-F_{is} \right)(s_{hom}-2s_{het})$. The fixation probability of the mutant allele with initial frequency $q_{0}=\frac{1}{2N_{e}}$, $u\left( \frac{1}{2N_{e}} \right)$, can be numerically calculated according to equation (B1). A Mathematica notebook using *NIntegrate* [] function is provided in a separate document to calculate the integral part of equation (B1) (Wolfram, 1996).

**Appendix C: A program in C for calculating fixation probability based on equations (8) and (10)**

#include <stdio.h>

#include <math.h>

#include <stdlib.h>

#define so 0.02 /* gametic selection coeffcient in ovule*/

#define sp 0.02 /* gametic selection coeffcient in pollen*/

#define t -0.03 /* homozygotic selection coefficent*/

#define h -0.02 /* heterozygotic selection coefficient */

#define alpha 1.0 /* selfing rate */

#define N 30.0 /* effective population size */

double factor(n)

int n;

{

int result;

if(n==0)

result=1;

else {

result=n*factor(n-1);

}

return(result);

}

double phiv(a,b) /* function of PHI value */

double a,b;

{

double result;

result=0.0;

result=1.0+pow(a,2.0)/factor(3)+pow(a,4.0)/factor(5)+pow(a,6.0)/factor(7)+pow(a,8.0)/factor(9)

+b*(1.0+a)*(1.0/6.0+2.0*pow(a,2.0)/factor(5)+3.0*pow(a,4.0)/factor(7)+4.0*pow(a,6.0)/factor(9)) +b*b*(7.0/factor(5)+2.0*a/factor(5)+69.0*pow(a,2.0)/factor(7)+6.0*pow(a,3.0)/factor(7)+282*pow(a,4.0)/factor(9)+12.0*pow(a,5.0)/factor(9)) +b*b*b*(27.0/factor(7)+27.0*a/factor(7)+ 348.0*a*a/factor(9)+204.0*a*a*a/factor(9)) +pow(b,4.0)*(321.0/factor(9)+132.0*a/factor(9)) +pow(b,5.0)*2265.0/factor(11);

return(result);

}

int main()

{

double a1,b1,a2,b2,gama1,gama2,A,B,s,F,S,T,kvratio,fxrate;

FILE *ptr;

char *pps;

ptr=fopen("thepredict_result","a");

pps="%9.7f%s";

a1=0.0;

b1=0.0;

a2=0.0;

b2=0.0;

gama1=0.0;

gama2=0.0;

A=0.0;

B=0.0;

kvratio=0.0;

fxrate=0.0;

s=0.0;

S=0.0;

T=0.0;

F=0.0;

F=alpha/(2.0-alpha);

s=so+sp;

S=-0.5*(1.0-F)*s+(1.0-F)*h+F*t;

T=(1.0-F)*(t-2.0*h);

/* fixation probability */

a1=(2.0*N-1.0)*S;

b1=2.0*(N-1.0)*T;

a2=2.0*N*S;

b2=2.0*N*T;

gama1=phiv(a1,b1)/phiv(a2,b2);

A=exp(2.0*(2.0*N-1.0)*S+2.0*(N-1.0)*T)-(1.0-1.0/(2.0*N))*gama1*exp((4.0*N-1.0)*S+(2.0*N-1.0)*T);

gama2=phiv(S,T/(2.0*N))/phiv(a2,b2);

B=exp(2.0*S)-gama2*exp((2.0*N+1.0)*S+N*T)/(2.0*N);

fxrate=A/B;

/* K/V ratio */

a1=N*S;

b1=N*T;

kvratio=exp(4.0*a1+2.0*b1)-(2.0*a1+4.0*b1/3.0)*exp(2.0*a1+b1)/phiv(2.0*a1,2.0*b1);

printf("fxrate=%f, kvratio=%f\n",fxrate, kvratio);

/* store results */

fprintf(ptr,pps,(double)N,",");

fprintf(ptr,pps,so,",");

fprintf(ptr,pps,sp,",");

fprintf(ptr,pps,t,",");

fprintf(ptr,pps,h,",");

fprintf(ptr,pps,alpha,",");

fprintf(ptr,pps,fxrate,",");

fprintf(ptr,pps,kvratio,"\n");

fclose(ptr);

}

**Appendix D: A program in C for Monte Carlo simulations**

#include<stdio.h>

#include<math.h>

#include<stdlib.h>

#include<time.h>

#define vv 100 /* for calculating mean and sd of fixation rate */

#define CS 10000 /* replicate numbers */

#define NN 30 /* population size */

#define so 0.02 /* selection coefficient in ovules*/

#define sp 0.02 /* selection coefficient in pollen */

#define tt 0.03 /* selection coefficient in genotype aa */

#define hh 0.02 /* selection coefficient in Aa */

#define alpha 0.90 /* selfing rate */

void nrerror(error_text)

char error_text[];

/* NumericalRecipes standard error handler*/

{

void exit();

fprintf(stderr,"Number Recipes run_time error...\n");

fprintf(stderr,"%s\n", error_text);

fprintf(stderr,"...now exiting to system...\n");

exit(1);

}

/* random number */

#define M1 259200

#define IA1 7141

#define IC1 54773

#define RM1 (1.0/M1)

#define M2 134456

#define IA2 8121

#define IC2 28411

#define RM2 (1.0/M2)

#define M3 243000

#define IA3 4561

#define IC3 51349

double ran1(long *idum)

{

static long ix1, ix2,ix3;

static double r[98];

double temp;

static int iff=0;

int j;

void nrerror();

if(*idum<0||iff==0){

iff=1;

ix1=(IC1-(*idum)) % M1;

ix1=(IA1*ix1+IC1) % M1;

ix2=ix1%M2;

ix1=(IA1*ix1+IC1) % M1;

ix3=ix1 % M3;

for(j=1;j<=97;j++){

ix1=(IA1*ix1+IC1) % M1;

ix2=(IA2*ix2+IC2) % M2;

r[j]=(ix1+ix2*RM2)*RM1;

}

*idum=1;

}

ix1=(IA1*ix1+IC1) % M1;

ix2=(IA2*ix2+IC2) % M2;

ix3=(IA3*ix3+IC3) % M3;

j=1+((97*ix3)/M3);

if(j>97||j<1) nrerror("RAN1: This can not happen");

temp=r[j];

r[j]=(ix1+ix2*RM2)*RM1;

return temp;

}

void main()

{

long idum;

int i,j,v,k,kk;

double fx,fxrate, fxpa,pAAs,pAas,paas,pAAr,pAar,paar,wo,wp,w,x,c0,mfxrate,vfxrate,pAA[3],pAa[3],paa[3],pA[3],pa[3],fre[3],nzygote[3];

char *pps;

FILE *ptr;

pps="%10.8f%s";

srand((unsigned)time(NULL));

idum=0;

ptr=fopen("simureult","a");

mfxrate=0.0;

vfxrate=0.0;

for(kk=1;kk<=vv;kk++){

c0=0.0;

fxpa=0.0;

fx=0.0;

fxrate=0.0;

printf("kk=%d\n",kk);

for(i=0;i<=CS;i++){

for(j=0;j<=2;j++){

pAA[j]=0.0;

pAa[j]=0.0;

paa[j]=0.0;

pA[j]=0.0;

pa[j]=0.0;

}

pAA[1]=1.0-1.0/(double)NN; /* initial genotype and gene frequencies */

pAa[1]=1.0/(double)NN;

paa[1]=0.0;

pA[1]=1.0-0.5/(double)NN;

pa[1]=0.5/(double)NN;

pAAs=0.0;

pAas=0.0;

paas=0.0;

pAAr=0.0;

pAar=0.0;

paar=0.0;

wo=0.0;

wp=0.0;

w=0.0;

x=0.0;

do{

/* selfing part gametic selection */

pAAs=pAA[1]+0.25*(1.0+0.5*(so+sp))*pAa[1];

pAas=0.5*pAa[1];

paas=paa[1]+0.25*(1.0-0.5*(so+sp))*pAa[1];

/* random mating part gametic selection */

wo=1.0-so*pa[1];

wp=1.0-sp*pa[1];

pAAr=pA[1]*pA[1]/(wo*wp);

pAar=pa[1]*pA[1]*(1.0-sp)/(wo*wp)+pA[1]*pa[1]*(1.0-so)/(wo*wp);

paar=pa[1]*pa[1]*(1.0-so)*(1.0-sp)/(wo*wp);

/* combined genotypes */

pAA[2]=alpha*pAAs+(1.0-alpha)*pAAr;

pAa[2]=alpha*pAas+(1.0-alpha)*pAar;

paa[2]=alpha*paas+(1.0-alpha)*paar;

/* zygotic selection */

w=1.0+hh*pAa[2]+tt*paa[2];

pAA[2]=pAA[2]/w;

pAa[2]=(1.0+hh)*pAa[2]/w;

paa[2]=(1.0+tt)*paa[2]/w;

c0=pAA[2]+pAa[2]+paa[2];

pAA[2]=pAA[2]/c0;

pAa[2]=pAa[2]/c0;

paa[2]=paa[2]/c0;

/* printf("pAA=%f,pAa=%f,paa=%f\n",pAA[2],pAa[2],paa[2]); */

/* genetic drift: sampling */

idum=-(long)rand();

for(j=0;j<=2;j++) { fre[j]=0.0;nzygote[j]=0.0;}

fre[0]=0.0;

fre[1]=fre[0]+pAA[2]+0.5*pAa[2];

fre[2]=1.0;

x=0.0;

for (v=1;v<=(2*NN);v++){

x=ran1(&idum);

for(k=1;k<=2;k++){

if ((x>fre[k-1])&&(x<=fre[k]))

nzygote[k]+=1.0;

}

}

c0=nzygote[1]+nzygote[2];

pA[2]=nzygote[1]/c0;

pa[2]=nzygote[2]/c0;

/* geneotype freq */

pAA[2]=pA[2]*pA[2]+pa[2]*pA[2]*alpha/(2.0-alpha);

pAa[2]=2.0*pA[2]*pa[2]*(1.0-alpha/(2.0-alpha));

paa[2]=1.0-pAA[2]-pAa[2];

pAA[1]=pAA[2];

pAa[1]=pAa[2];

paa[1]=paa[2];

pA[1]=pA[2];

pa[1]=pa[2];

fxpa=pa[2];

}while((pA[2]>=0.0001)&&(pA[2]<=0.999)); /* reaching steady state */

if(fxpa==1.0) fx+=1.0;

} /* i loop for CS */

fxrate=fx/(double)CS;

mfxrate+=fxrate;

vfxrate+=fxrate*fxrate;

}

c0=(double)vv;

mfxrate=mfxrate/c0;

vfxrate=(vfxrate-c0*mfxrate*mfxrate)/(c0-1.0);

vfxrate=sqrt(vfxrate);

printf("mfxrate=%f,vfxrate=%f\n",mfxrate,vfxrate);

fprintf(ptr,pps,(double)CS,",");

fprintf(ptr,pps,(double)NN, ",");

fprintf(ptr,pps,alpha, ",");

fprintf(ptr,pps,so,",");

fprintf(ptr,pps,sp,",");

fprintf(ptr,pps,tt, ",");

fprintf(ptr,pps,hh,",");

fprintf(ptr,pps,mfxrate,",");

fprintf(ptr,pps,vfxrate,"\n");

fclose(ptr);

}

**Table S1**. The fixation probability of a mutant allele derived from Wright’s and Kimura’s methods under phase variation in selection

| Selection | Expressed genes | Wright’s method | Kimura’s method |
| --- | --- | --- | --- |
| Gametophytic selection  $s_{hap}\neq0,s_{hom}=s_{het}=0$ | Gametophyte-specific genes | $u(\frac{1}{2N_{e}})=\frac{\exp\left( (1-F_{is})s_{hap} \right)-1}{\exp\left( 2(2N_{e}-1)(1-F_{is})s_{hap} \right)-1}$ | $u\left( \frac{1}{2N_{e}} \right)=\frac{\exp\left( (1-F_{is})s_{hap} \right)-1}{\exp\left( 4N_{e}(1-F_{is})s_{hap} \right)-1}$ |
| Sporophytic selection (additive)  $s_{hap}=0,s_{hom}=2s_{het}(\neq0)$ | Sporophyte-specific genes | $u(\frac{1}{2N_{e}})=\frac{1-\exp\left( -2(1+F_{is})s_{het} \right)}{1-\exp\left( -2(2N_{e}-1)(1+F_{is})s_{het} \right)}$ | $u(\frac{1}{2N_{e}})=\frac{1-\exp\left( -2(1+F_{is})s_{het} \right)}{1-\exp\left( -4(1+F_{is})N_{e}s_{het} \right)}$ |
| Biphasic selection(additive)  $s_{hap}\neq0,s_{hom}=2s_{het}(\neq0)$ | Pleiotropic genes | $u(\frac{1}{2N_{e}})=\frac{1-\exp\left( (1-F_{is})s_{hap}-2(1+F_{is})s_{het} \right)}{1-\exp\left( (2N_{e}-1)\left( (1-F_{is})s_{hap}-2(1+F_{is})s_{het} \right) \right)}$ | $u(\frac{1}{2N_{e}})=\frac{1-\exp\left( (1-F_{is})s_{hap}-2(1+F_{is})s_{het} \right)}{1-\exp\left( {2N}_{e}\left( (1-F_{is})s_{hap}-2(1+F_{is})s_{het} \right) \right)}$ |
| Biphasic selection (nonadditive)  $s_{hap}\neq0,s_{hom}\neq2s_{het}\left( \neq0 \right)$,  $\alpha=1-\frac{s_{hom}}{s_{hap}-2s_{het}+s_{hom}}$ | Pleiotropic genes | $u(\frac{1}{2N_{e}})=\frac{\exp\left( 2\left( N_{e}-1 \right)T \right)-\left( 1-\frac{1}{2N_{e}} \right)\gamma_{1}\exp\left( \left( 2N_{e}-1 \right)T \right)}{1-\frac{1}{2N_{e}}\gamma_{2}\exp\left( 2N_{e}T \right)}$, $\gamma_{1}=\frac{\psi\left( 0,2\left( N_{e}-1 \right)T \right)}{\psi\left( 0,2N_{e}T \right)}$ , $\gamma_{2}=\frac{1}{\psi\left( 0,2N_{e}T \right)}$ | $u\left( \frac{1}{2N_{e}} \right)=\frac{erf(\sqrt{\frac{T}{2N_{e}}})}{erf(\sqrt{2N_{e}T})}$ ,  $\mathrm{erf} \left( z \right)=\frac{2}{\sqrt{\pi}}\int_{0}^{z} e^{-t^{2}}dt$ |

**Figures S1 and S2**

**Figure S1**. Examples of fixation probabilities under different cases of gametophytic and sporophytic selection. The results are calculated from equation (9) in the main text. The effective population size is set as $N_{e}$=15. The simulated cases are the gametophytic selection only ($s_{O}=s_{P}$=0.02, $s_{hom}$=0.00, and $s_{het}$=0.00 for the deleterious mutant allele; $s_{O}=s_{P}$=-0.02, $s_{hom}$=0.00, and $s_{het}$=0.00 for the favorable mutant allele), the sporophytic selection ($s_{O}=s_{P}$=0.00, $s_{hom}$=-0.03, and $s_{het}$=-0.02 for the deleterious mutant allele; $s_{O}=s_{P}$=0.0, $s_{hom}$=0.03, and $s_{het}$=0.02 for the favorable mutant allele), the antagonistic selection ($s_{O}=s_{P}$=-0.02, $s_{hom}$=-0.03, and $s_{het}$= -0.02; $s_{O}=s_{P}$=0.02, $s_{hom}$=0.03, and $s_{het}$=0.02) and the synergistic selection ($s_{O}=s_{P}$=0.02, $s_{hom}$=-0.03, and $s_{het}$=-0.02 for the deleterious mutant allele; $s_{O}=s_{P}$=-0.02, $s_{hom}$=0.03, and $s_{het}$=0.02 for the favorable mutant allele).

**Figure S2**. Examples of the $K/\mu$ ratios under different cases of gametophytic and sporophytic selection. The results are calculated from equation (10) in the main text. The effective population size is set as $N_{e}$=15. The simulated cases are the gametophytic selection only ($s_{O}=s_{P}$=0.02, $s_{hom}$=0.00, and $s_{het}$=0.00 for the deleterious mutant allele; $s_{O}=s_{P}$=-0.02, $s_{hom}$=0.00, and $s_{het}$=0.00 for the favorable mutant allele), the sporophytic selection ($s_{O}=s_{P}$=0.00, $s_{hom}$=-0.03, and $s_{het}$=-0.02 for the deleterious mutant allele; $s_{O}=s_{P}$=0.0, $t$=0.03, and $s_{het}$=0.02 for the favorable mutant allele), the antagonistic selection ($s_{O}=s_{P}$=-0.02, $s_{hom}$=-0.03, and $s_{het}$= -0.02; $s_{O}=s_{P}$=0.02, $s_{hom}$=0.03, and $s_{het}$=0.02) and the synergistic selection ($s_{O}=s_{P}$=0.02, $s_{hom}$=-0.03, and $s_{het}$=-0.02 for the deleterious mutant allele; $s_{O}=s_{P}$=-0.02, $s_{hom}$=0.03, and $s_{het}$=0.02 for the favorable mutant allele).
